# Pap Cytology and Human Papillomavirus (HPV) Genotypes in Western Romania: A Retrospective Analysis

**DOI:** 10.7759/cureus.65720

**Published:** 2024-07-30

**Authors:** Sebastian Ciurescu, Sas Ioan, Florin I Gorun, Croitoru Pop Madalina, Diana Ilas, Tomescu Larisa

**Affiliations:** 1 Obstetrics and Gynecology, Victor Babeş University of Medicine and Pharmacy, Timisoara, ROU; 2 Obstetrics and Gynecology, Timisoara Clinical Municipal Emergency Hospital, Timisoara, ROU; 3 Medical Semiology, Victor Babeş University of Medicine and Pharmacy, Timisoara, ROU

**Keywords:** cytology grading, cervical cancer screening, human papillomavirus, pap smear, cytology

## Abstract

Background and objective

Vigorous proactive measures are needed to combat cervical cancer, linked to the human papillomavirus (HPV). HPV genotyping aids in identifying high-risk strains, enabling targeted screening and risk assessment. This informs treatment decisions, reducing cervical cancer cases. In light of this, we conducted a retrospective analysis of Pap cytology and HPV genotypes to assess western Romania’s new screening program. Understanding HPV prevalence, genotype correlations, and risk factors will help refine risk stratification models and enhance public health strategies.

Methodology

This retrospective study analyzed Pap smears from 195 patients with a mean age of 40 years [standard deviation (SD): 12 years], with a peak of incidence between 25 and 30 years. The patient cohort, with equal representation from urban and rural areas, comprised sexually active women of reproductive age presenting to the Gynaecology Ambulatory of the Municipal Emergency Clinical Hospital Timișoara over two years. Patients not sexually active and those in menopause were excluded. HPV genotyping was done on 67 patients. Data were analyzed using JASP, employing descriptive statistics, frequency tables, contingency tables, chi-squared test, odds ratio, and Fisher's exact test.

Results

Among 195 patients undergoing Pap smear tests, the most prevalent finding was negative (77.95%), indicating no abnormal cells detected. A smaller proportion of patients exhibited low-grade squamous intraepithelial lesions (LSIL, 11.28%) or atypical squamous cells of undetermined significance (ASC-US, 8.72%) findings. High-grade lesions (HSIL, ASC-H) were rare. For cytology grading, Category II (CII) was the most prevalent (75.90%), followed by Category III (CIII, 24.10%). HPV genotyping was performed on a subset of patients (n=67). Among them, Type 16 was the most frequent (28.36%), followed by Other high-risk types (20.89%), Type 16 (28.35%), and Type 18 (13.43%). HPV testing was not performed for 65.64% of the patients. Overall, the study suggests that a significant majority of the patients had normal Pap smears. However, a small proportion did have abnormal findings, including HPV infection. These findings highlight the importance of Pap smear screening for early detection of cervical abnormalities.

Conclusions

Our study from western Romania highlights the importance of HPV testing and Pap cytology in cervical cancer prevention. HPV testing is a potent tool for identifying high-risk women, and when combined with Pap cytology, it provides a more comprehensive screening strategy. Our research also revealed a diverse HPV genotype distribution, suggesting the need for broader-spectrum vaccines like the nonavalent vaccine. Despite our study’s limitations, our findings underscore the need for including HPV testing in national screening guidelines. Future research should focus on larger studies and the cost-effectiveness of broader-spectrum vaccines. Implementing policies based on these findings could lead to more effective cervical cancer prevention in western Romania.

## Introduction

Cervical cancer, a preventable disease affecting millions worldwide, requires strong proactive measures to combat it. The human papillomavirus (HPV) is the leading culprit, making prevention strategies like HPV genotyping a powerful tool. Genotyping helps identify women with high-risk HPV strains, allowing for targeted screening and risk stratification. This valuable data informs treatment decisions for precancerous lesions, ultimately leading to a significant reduction in cervical cancer cases. In the wake of the launch of a new national cervical cancer screening program in western Romania, we conducted a retrospective analysis of Pap cytology results and corresponding HPV genotypes. We believe such an analysis would provide a comprehensive picture of the region's HPV landscape and the effectiveness of the new program.

By understanding the prevalence of different HPV strains, the correlation between Pap results and HPV types, and the risk factors associated with specific genotypes, we can refine risk stratification models, improve early detection, and ultimately inform more effective public health strategies for western Romania. The recent implementation of a national cervical cancer screening program in western Romania presents a compelling opportunity to investigate the regional landscape of HPV and its associated risks for women's health. This study aims to provide a retrospective analysis of Pap cytology results and corresponding HPV genotypes. By meticulously examining this data, we aim to achieve a comprehensive understanding of HPV prevalence, its correlation with Pap cytology findings, and the subsequent refinement of risk stratification models for this specific population.

Our primary objective is to elucidate the distribution and prevalence of various HPV genotypes within the female population of western Romania, to gain crucial insights into the dominant HPV strains circulating in the region, allowing for focused vaccination efforts and identification of populations at higher risk of HPV infection and potential cervical cancer development. Furthermore, we will analyze the correlation between Pap cytology results and identified HPV types. This analysis will assess the effectiveness of the newly implemented screening program in detecting precancerous lesions associated with high-risk HPV strains. Evaluating the program's efficacy in this specific context will be instrumental in informing future optimization strategies.

Finally, we will delve into the association between specific HPV genotypes and cytological abnormalities identified in Pap smears. This information will be critical for refining risk stratification models for western Romania. By understanding how different HPV strains correlate with varying degrees of cellular changes, we can tailor clinical management strategies for women, to ensure they receive the most appropriate level of follow-up and treatment based on their risk profile, ultimately leading to improved patient outcomes.

## Materials and methods

We adopted a retrospective study design, meticulously analyzing Pap smears from 195 patients. The mean age of these patients was 40 years [standard deviation (SD): 12], with a notable peak between 25 and 30 years. The provenance of the patients was almost equally distributed between urban and rural areas, providing a balanced representation of the population. We included all sexually active women of reproductive age presenting to the Gynaecology Ambulatory of the Municipal Emergency Clinical Hospital Timișoara over two years. Patients who had not initiated their sexual activity, those in menopause, or did not want to participate in the study were excluded, to maintain the focus of our research. We aimed to determine whether Pap smears alone are sufficient for detecting all HPV cases or if additional HPV genotyping is necessary. We addressed this via HPV genotyping on a subset of 67 patients who had undergone screening as part of a national program. The results were meticulously input into Excel, forming a comprehensive database.

This data was then statistically investigated using the latest versions of JASP. Our statistical approach was multifaceted. We employed descriptive statistics, frequency tables, and contingency tables to provide a comprehensive overview of the data. Furthermore, we use the chi-square test to determine the statistical significance of our findings. Also, we calculated the odds ratio and performed the Fisher's test to provide further insights into the relationships within our data. Moreover, we conducted a correlation analysis followed by a logistic regression analysis to explore a possible association between cytology results and HPV genotyping and to understand how HPV genotypes influence the probability of different Pap smear grades.

This rigorous and methodical approach ensured that our study was both comprehensive and robust, providing valuable insights into Pap cytology and HPV genotypes in western Romania. Our findings contribute to the broader understanding of these topics and pave the way for future research in this area.

## Results

**Figure 1:**
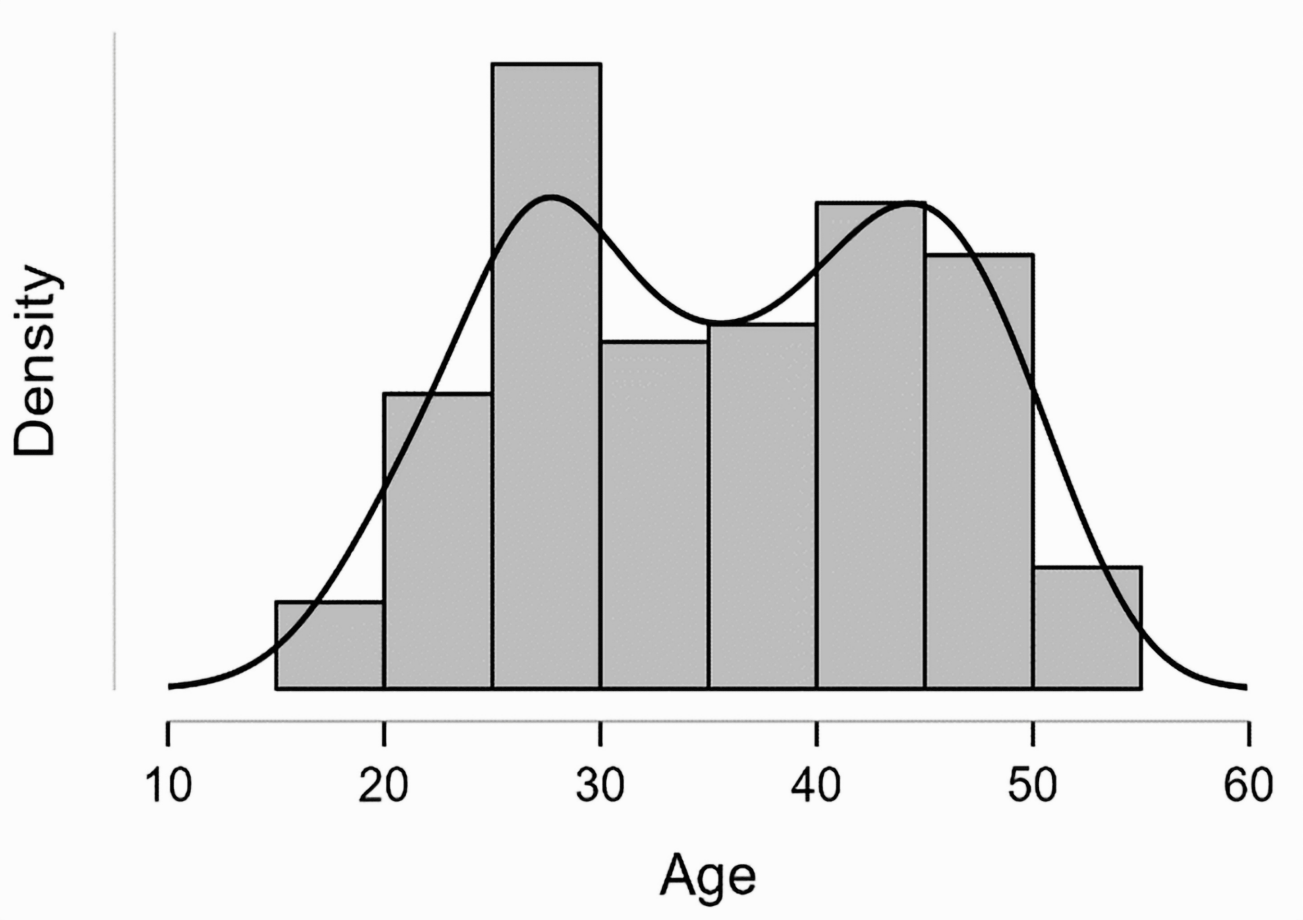
Distribution of patients by age group

We provide a detailed examination of the characteristics of the studied population here. This population comprised 195 patients, all of whom were sexually active women of reproductive age presenting at the Gynaecology Ambulatory of the Municipal Emergency Clinical Hospital Timișoara over two years. As shown in Table 1, the mean age of these patients was 40 years (SD: 12 years). A significant peak was observed in the age distribution between 25 and 30 years, as displayed in Figure 1. The geographical distribution of the patients was balanced, with almost equal representation from urban and rural areas, ensuring that the study was not biased towards any particular demographic and provided a comprehensive overview of the population in western Romania.

**Table 1:** Age distribution in the cohort CI: confidence interval

Descriptive Statistics	Age, Years
Mean	40.072
Std. Error of Mean	0.905
95% CI Mean Upper	41.857
95% CI Mean Lower	38.287
Std. Deviation	12.640
Minimum	16.000
Maximum	78.000

**Figure 2:**
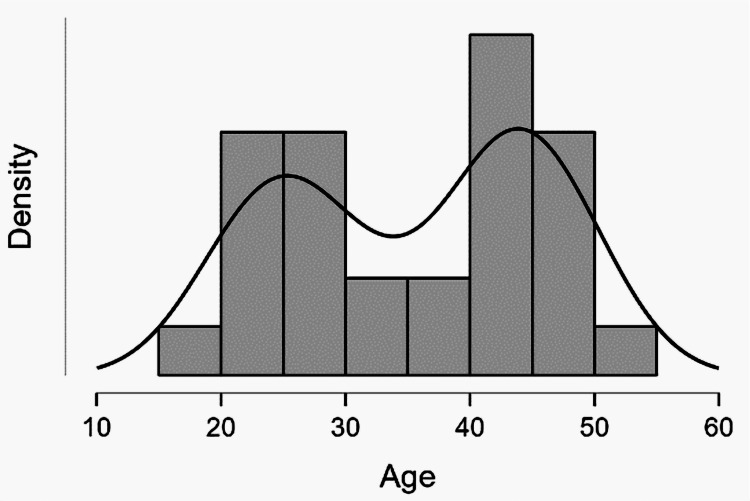
Distribution of patients from rural areas by age group

**Figure 3:**
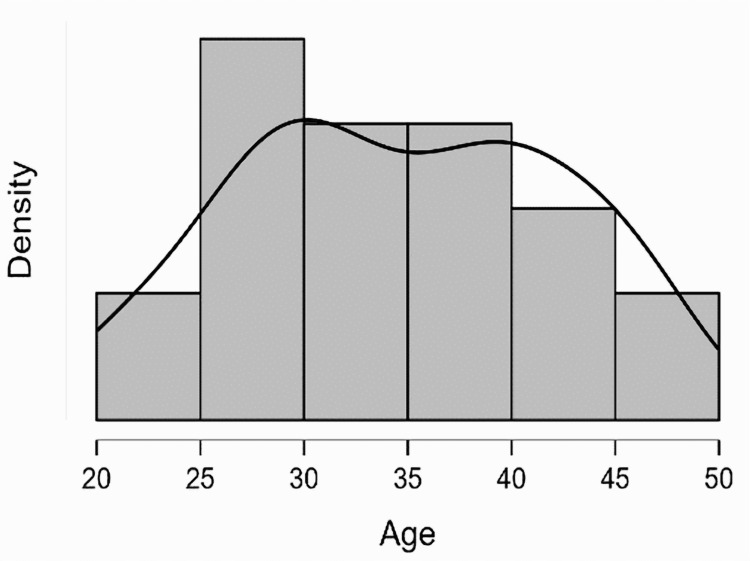
Distribution of patients from urban areas by age group

We employed stringent inclusion criteria for our study, focusing only on sexually active women of reproductive age. We excluded patients who had not initiated their sexual activity or did not want to participate in the study, ensuring that our research was focused and relevant. In our subset of 67 patients, we conducted HPV genotyping and observed a unique age distribution, as shown in Table 2. The average age in this group was 36 years (SD: 10 years). Notably, we identified two distinct peaks in the age distribution at the intervals of 25-30 years and 40 years, as shown in Figures 2-3. The first peak, within the 25-30 years interval, predominantly comprised women from urban areas. This could be attributed to these women having better access to health campaigns and more comprehensive information about the risks of HPV. The second peak at 40 years was more characteristic of women from rural areas. This could be due to the differences in the availability and reach of health campaigns and information on HPV in these areas.

**Table 2:** Age distribution of HPV genotyped patients CI: confidence interval: HPV: human papillomavirus

Descriptive Statistics	Age, Years
Mean	36.851
95% CI Mean Upper	39.438
95% CI Mean Lower	34.263
Std. Deviation	10.608
Minimum	19.000
Maximum	63.000

Pap smear tests were conducted on all 195 patients included in the study, and the most prevalent finding was negative, indicating that no abnormal cells were detected, which accounted for a substantial 77.949% of the total cases. This suggests that a significant majority of the patients in our study did not exhibit any abnormal cells in their Pap smear tests. Low-grade squamous intraepithelial lesion (LSIL) was the second most common finding (22 cases, 11.282%). This indicates that a smaller, yet notable, proportion of patients exhibited low-grade lesions in their Pap smear tests. Atypical squamous cells of undetermined significance (ASC-US) were noted in 17 instances (8.718%). This suggests that a certain percentage of patients had atypical squamous cells, the significance of which could not be determined.

High-grade squamous intraepithelial lesions (HSIL) were identified in a mere three cases, making up 1.538% of the total. This indicates that a small number of patients exhibited high-grade lesions in their Pap smear tests. Lastly, there was one case classified as ASC-H (atypical squamous cells - cannot exclude HSIL), accounting for 0.513% of the total. This suggests that a very small number of patients had atypical squamous cells that could not exclude a high-grade lesion. These findings are presented in Table 3.

**Table 3:** Pap smear test results in the cohort ASC-H: atypical squamous cells – cannot exclude HSIL; ASC-US: atypical squamous cells of undetermined significance; HSIL: high-grade squamous intraepithelial lesion; LSIL: low-grade squamous intraepithelial lesion

Pap Test Results	Frequency	Percentage	Cumulative Percentage
ASC-H	1	0.51%	0.51%
ASC-US	17	8.71%	9.23%
HSIL	3	1.53%	10.76%
LSIL	22	11.28%	22.05%
Negative	152	77.94%	100.00%
Total	195	100.00%	

In the process of discerning cellular modifications, the results of the Pap smear tests were also stratified according to a specific grading system. As shown in Table 4, the subsequent data elucidates the distribution of these results: Cytology II (CII): this category comprised 148 cases, which constituted 75.897% of the total. This significant proportion indicates a high prevalence of CII in our sample. Cytology III (CIII): this category included 47 cases, making up 24.103% of the total. Although less prevalent than CII, CIII still represents a notable portion of our sample. These findings contribute significantly to our understanding of the prevalence and patterns of cervical abnormalities.

**Table 4:** Cytology grading in the cohort CII: Class II; CIII: Class III

Cytology	Frequency	Percentage	Valid Percentage	Cumulative Percentage
CII	148	75.89%	75.89%	75.89%
CIII	47	24.10%	24.10%	100.00%
Total	195	100.00%		

Of the 195 patients, only 67 had undergone HPV genotyping. We drew valuable insights into the prevalence of different HPV types within the subset of patients who were tested, as described in Table 5. The genotyping results were systematically classified into four categories: (1) Other high-risk types encompassed 14 instances, constituting 7.179% of the aggregate frequency and 20.896% of the valid percentage; (2) Type 16 incorporated 19 instances, accounting for 9.744% of the aggregate frequency and 28.358% of the valid percentage; (3) Type 18 was responsible for nine instances, signifying 4.615% of the aggregate frequency and 13.433% of the valid percentage; and (4) Negative, indicative of no HPV detection, comprised 25 instances, which constituted 12.821% of the aggregate frequency and made up the residual 37.313% of the valid percentage. It is critical to highlight that 128 patients were not subjected to testing, which represents 65.641% of the total sample size of 195 patients. 

**Table 5:** Results of HPV genotyping HPV: human papillomavirus

Type	Frequency	Percentage	Valid Percentage	Cumulative Percentage
Other High-Risk Types	14	7.17%	20.89%	20.89%
Type 16	19	9.74%	28.35%	49.25%
Type 18	9	4.61%	13.43%	62.68%
Negative	25	12.82%	37.31%	100.00%
Missing	128	65.64%		
Total	195	100.00%		

Our investigation was centered on the examination of the contingency relationship between cytological findings and HPV genotyping outcomes. As shown in Table 6, we utilized a chi-squared test as a statistical tool to ascertain the association between these parameters, in conjunction with the computation of odds ratios. The findings from our study were indeed encouraging. We ascertained that the likelihood of a patient being classified with CIII escalates by a factor of 16 if they also test positive in the HPV genotyping test. This observation underscores the advantages of implementing a robust national program for HPV screening. The p-value obtained from the chi-squared test was hugely significant, registering at 9.13 × 10−8, thereby reinforcing the validity of our findings.

**Table 6:** Contingency table on cytology and HPV genotyping results CII: Class II; CIII: Class III; HPV: human papillomavirus

HPV Status		Cytology		
		CII	CIII	Total
Negative	Count	16.00	9.00	25.00
	% of total	23.88%	13.43%	37.31%
Positive	Count	4.00	38.00	42.00
	% of total	5.97%	56.71%	62.68%
Total	Count	20.00	47.00	67.00
	% of total	29.85%	70.14%	100.00%

We conducted a comprehensive correlation study between cytology grading and various HPV types. The findings revealed a moderate correlation, with each HPV type associated with a unique p-value, highlighting the statistical significance of our results. The strength and significance of the correlations between cytology grades and different HPV types are as follows: specifically, the Pearson's r values are 0.173 for Type 16, 0.196 for Type 18, and 0.294 for Other high-risk types. The corresponding p-values are 0.0178 for Type 16, 0.0126 for Type 18, and a value of 0.020 for Other high-risk types. These numerical values underscore a weak correlation between cytology grading and various HPV types. Other high-risk types suggest a more noticeable linear relationship with cytology grading compared to Type 16 and 18. The low p-value of 0.020 indicates that this correlation is statistically significant and is less likely to have occurred by chance. These findings are presented in Table 7.

**Table 7:** Correlations between cytology grading and HPV types HPV: human papillomavirus

Variable	Cytology Grading	
1. Cytology Grading	Pearson's r	-
	P-value	-
2. Type 16	Pearson's r	0.173
	P-value	0.0178
3. Type 18	Pearson's r	0.196
	P-value	0.0126
4. Other High-Risk Types	Pearson's r	0.294
	P-value	0.020

We further analyzed the data by employing a logistic regression to determine the association between HPV genotypes and Pap smear test grading. The model fit statistics indicated a significant improvement (p<0.001) when including HPV genotypes (Type 16, Type 18, Other) compared to the null model. This suggests that HPV genotypes contribute to explaining the variation in Pap smear results. The coefficients table revealed a negative intercept (-0.511), representing the log odds of a positive Pap smear in the absence of HPV genotypes. However, the primary focus lies on the relative effects of HPV genotypes.

**Figure 4:**
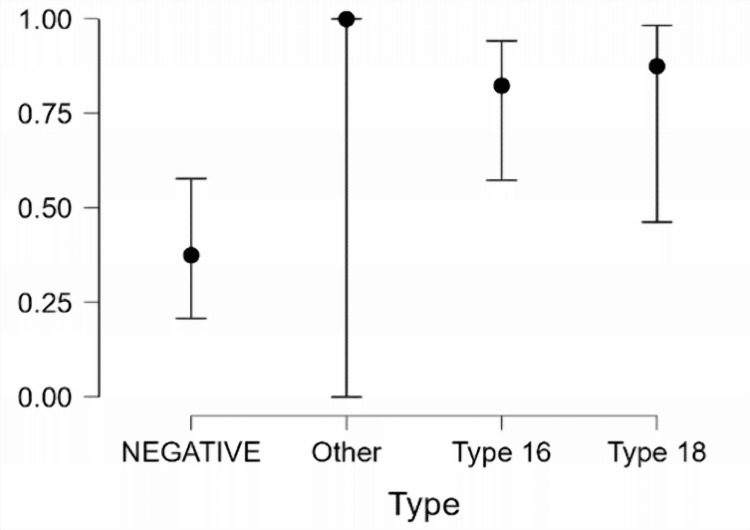
Estimates plots

Notably, Type (Other) lacked statistical significance (p=0.992), precluding a definitive conclusion regarding its association with Pap smear results. Conversely, both Type 16 (coefficient=2.051) and Type 18 (coefficient=2.457) displayed statistically significant positive coefficients (p<0.05) (Table 8). This indicates that compared to the baseline (no HPV genotypes), the presence of either Type 16 or Type 18 HPV genotypes increases the odds of having a positive Pap smear result. The findings are further corroborated by the estimates plots (Figure 4), which visually represent the predicted probability of a positive Pap smear for each HPV genotype. The graph demonstrates the highest probability for Type 18, followed by Type 16, and lastly, Other.

**Table 8:** Coefficients table

					Wald Test
	Estimate	Z	Wald Statistic	df	P
(Intercept)	-0.511	-1.212	1.468	1	0.226
Type (Other)	19.077	0.011	1.112 × 10^-4^	1	0.992
Type (Type 16)	2.051	2.688	7.223	1	0.007
Type (Type 18)	2.457	2.138	4.570	1	0.033

Thus, the logistic regression analysis provides compelling evidence that HPV genotypes, particularly Type 16 and Type 18, are positively associated with an increased likelihood of a positive Pap smear result (CIII). These findings highlight the potential role of HPV genotyping in refining cervical cancer screening strategies.

## Discussion

Our study from Western Romania contributes valuable insights to the understanding of HPV genotypes and their association with abnormal Pap smears in this under-studied region. We observed a high prevalence of HPV infection among women undergoing Pap cytology screening, mirroring patterns identified globally [1-5]. HPV 16 emerged as the most prevalent genotype, followed by HPV 18, and Other high-risk types. This distribution aligns with previous research, highlighting the dominance of HPV 16 as a major oncogenic factor across diverse populations [1,6]. Of note, the presence of high-risk HPV genotypes, particularly HPV 16 and 18, strongly correlated with abnormal Pap test results (ASC-US or worse), reinforcing the established role of HPV testing as a crucial tool for identifying women at heightened risk for developing cervical cancer and precancerous lesions [7,8]. Our study goes beyond simply confirming existing knowledge. By delving deeper into the specific HPV genotype distribution within our Romanian cohort, we contribute valuable data for regional public health initiatives.

The presence of the dominant HPV 16 indicates a need for broader vaccine coverage. Vaccines currently available in Romania, like the quadrivalent vaccine, may not offer sufficient protection against these prevalent non-16/18 hrHPV types [9,10]. Hence, adopting broader-spectrum vaccines such as the nonavalent vaccine could ensure more effective prevention of cervical cancer [11]. Our findings on HPV genotype distribution are consistent with several studies worldwide [11-15]. Similar to our results, previous research has shown a higher prevalence of HPV infection in women with abnormal Pap cytology compared to those with normal cytology [16,17]. This affirms the established synergy between Pap cytology and HPV testing for a more accurate and comprehensive cervical cancer screening strategy [17,18]. Our study also aligns with prior research on the clearance rates of different HPV genotypes. The faster clearance of HPV 16 and 18 than other types in our study aligns with other studies [18], highlighting the potential for HPV testing to not only identify women at risk but also potentially monitor clearance of infection over time.

It is important to acknowledge the limitations of our study. The retrospective design inherently restricted our ability to establish definitive causal relationships between HPV infection and abnormal Pap smears. Additionally, our study population was drawn from a specific geographic region in Romania, potentially limiting the generalizability of our findings to the broader Romanian population; A larger, prospective study with a more diverse population would be necessary to strengthen the generalizability of our results. Furthermore, our study did not explore factors influencing HPV infection rates, such as sexual behavior and vaccination status. Future research on these factors alongside HPV genotypes could provide a more comprehensive understanding of cervical cancer risk in western Romania.

Despite these limitations, our findings hold significant clinical relevance for patient management and optimizing cervical cancer screening programs in western Romania. The high prevalence of HPV infection and its strong association with abnormal Pap smears underscore the critical role of HPV testing alongside Pap cytology for cervical cancer screening in this region. We recommend including HPV testing in national cervical cancer screening guidelines. We also highlight the importance of promoting HPV vaccination programs, particularly vaccines targeting a broader range of HPV genotypes. The prevalence of HPV genotypes 52 and 58 suggests that current vaccines may not offer optimal protection for the Romanian population. Adoption of broader-spectrum vaccines could significantly enhance cervical cancer prevention efforts [13]. Also, our results support the recommendations for colposcopy with biopsy for women with persistent non-16/18 HPV infection identified through liquid-based cytology [17]. Implementing such protocols could ensure timely diagnosis and interventions for women at high risk of developing cervical cancer. 

Additionally, increasing awareness among healthcare providers regarding risk factors for HPV infection, such as young age and multiple sexual partners, can be instrumental in the early detection and management of cervical cancer [18]. By incorporating these findings into clinical practice and public health initiatives, we can move towards a more effective and targeted approach to cervical cancer prevention in western Romania. Future research efforts should focus on conducting larger, prospective studies to confirm our findings and explore additional factors influencing HPV infection rates in this region.

## Conclusions

This study sheds light on crucial aspects of cervical cancer prevention and management in western Romania. We confirm the value of HPV testing as a powerful tool to identify women at high risk for cervical cancer. By combining Pap cytology with HPV testing, a more accurate and comprehensive screening strategy is achieved compared to relying solely on Pap cytology. Our research goes beyond simply verifying existing knowledge. A deeper analysis of the specific HPV genotype distribution within our study population yields valuable insights for regional public health initiatives. 

The presence of HPV genotypes beyond the dominant HPV 16 suggests limitations in the current vaccination strategy. We recommend the adoption of broader-spectrum vaccines, such as the nonavalent vaccine, which could be instrumental in more effectively preventing cervical cancer in this region. The high prevalence of HPV infection and its strong correlation with abnormal Pap smears highlight the critical importance of including HPV testing alongside Pap cytology for cervical cancer screening in western Romania. This strengthens the case for incorporating HPV testing into national cervical cancer screening guidelines. Incorporating these findings into clinical practice and public health initiatives can help us reach a more effective and targeted approach to cervical cancer prevention and management in western Romania.
